# Endotoxin’s Impact on Organism: From Immune Activation to Tolerance and Beyond

**DOI:** 10.3390/jcm14186478

**Published:** 2025-09-14

**Authors:** Kacper Roszak, Konkonika Roy, Justyna Sobocińska, Paulina Spisz, Tomasz Jędrzejewski, Sylwia Wrotek

**Affiliations:** Department of Immunology, Faculty of Biological and Veterinary Sciences, Nicolaus Copernicus University in Toruń, 87-100 Toruń, Poland; konkonika.roy@doktorant.umk.pl (K.R.); j.sobocinska@umk.pl (J.S.); p.spisz@umk.pl (P.S.); tomaszj@umk.pl (T.J.)

**Keywords:** endotoxin, LPS, endotoxin tolerance, inflammation, sepsis, septic shock, endotoxemia, inflammasome, TLR4 signalling, tumor microenvironment

## Abstract

Endotoxin, a key component of Gram-negative bacterial membranes, remains a central focus in understanding host–pathogen interactions and immune modulation. In this review, we examine the multifaceted roles of endotoxin, with particular emphasis on how its structural variants modulate host immune recognition and inflammatory signalling. We explore the complex mechanisms by which endotoxin activates innate immune pathways and how these responses evolve under repeated or chronic exposure conditions. Special attention is given to the phenomenon of endotoxin tolerance, an adaptive reprogramming of immune cells that can profoundly alter inflammatory outcomes. While this tolerance serves as a protective mechanism against hyperinflammation, mounting evidence suggests that it may have a “dark side”, potentially impairing immune surveillance and promoting disease states such as cancer. We also discuss emerging clinical and therapeutic strategies that harness or counteract endotoxin-driven responses, from vaccine adjuvants to anti-sepsis interventions. By integrating recent findings from immunology, microbiology, and translational medicine, this review sheds light on the dual nature of endotoxin and its far-reaching implications for human health.

## 1. Introduction

Endotoxin, most commonly represented by lipopolysaccharide (LPS), is a key component of the outer membrane of Gram-negative bacteria. Upon bacterial infection, endotoxin is recognised by the host immune system, triggering a cascade of immune responses aimed at combating the invading pathogens. This includes the activation of various immune cells, the release of pro-inflammatory cytokines, and the initiation of adaptive immune responses [1].

Initially, the immune system mounts an acute response to LPS, aiming to eliminate bacteria. However, when the pathogen cannot be fully eradicated, chronic low-grade inflammation may develop. This immune state has been implicated in the development of various chronic diseases, including atherosclerosis, insulin resistance, and even cancer [2,3,4]. Alternatively, persistent exposure to endotoxin can, in some cases, lead to endotoxin tolerance (ET). In this adaptive state, immune cells become less responsive to LPS stimulation, thereby limiting excessive inflammation and protecting host tissues from collateral damage. This duality between chronic inflammation and immune desensitization illustrates the complexity of host–pathogen interactions. It establishes a dynamic feedback loop in which adaptations by one party drive counter-adaptations in the other. For instance, as bacteria evolve structural modifications to LPS that enhance immune evasion, the host may, in turn, refine its sensing and regulatory mechanisms to maintain immune equilibrium. This ongoing molecular arms race determines the outcome of individual infections and influences the long-term co-evolution of microbial pathogens and their hosts.

This review aims to clarify the dualistic nature of endotoxin, from immune activation to immune suppression, and highlight its significance in health and disease by synthesising recent advances in endotoxin signalling, tolerance development, immune adaptation, and exploring potential clinical applications and therapeutic targets.

## 2. Endotoxin—A Pivotal Pro-Inflammatory Factor in Gram-Negative Bacterial Pathogenesis

LPS is a classic example of a Pathogen-Associated Molecular Pattern (PAMP), a conserved molecule found in many pathogens and recognised by the innate immune system. It is specifically found in the outer membrane of Gram-negative bacteria [3]. Gram-negative bacteria constitute a significant portion of microorganisms responsible for widespread illnesses. For example, *Escherichia coli* is one of the most common human pathogens, causing urinary tract infections, gastrointestinal diseases, and respiratory tract infections [5]. *Klebsiella pneumoniae* is another example of a Gram-negative bacterial infection, which can lead to severe conditions such as pneumonia, urinary tract infections, and bloodstream infections [6]. Additionally, *Pseudomonas aeruginosa* is commonly associated with hospital-acquired infections, causing skin infections, wounds, respiratory tract infections, and urinary tract infections, particularly in patients with reduced immunity [7]. These examples underscore the importance of Gram-negative bacteria as a significant problem in various contexts. Therefore, it is necessary to recognise all aspects of the hosts’ response to endotoxin.

**Figure 1:**
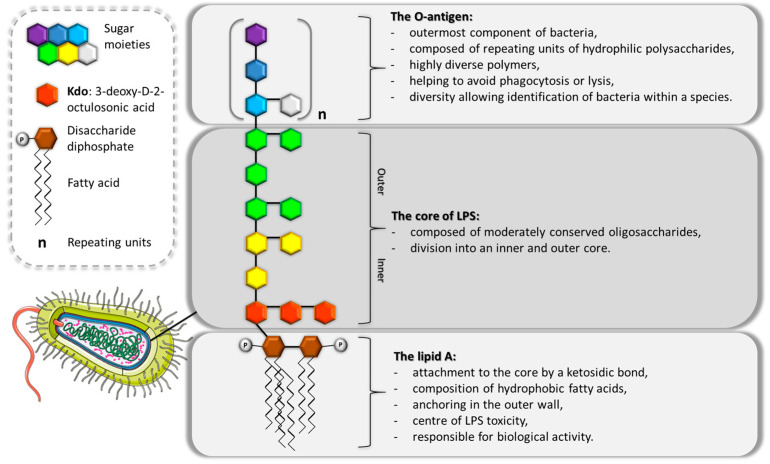
The general structure of lipopolysaccharide [9,10,11,12,13].

Many years of research have led to our understanding of the structure of LPS. It is known that endotoxin consists of three functionally, biosynthetically and structurally independent parts: the O-antigen, core and lipid A [8] (Figure 1).

For most Gram-negative bacteria, it is ordered and coherent, but there are exceptions, e.g., environmental strains [9,14], *Neisseria* [15], *Haemophilus* [16], *Xanthomonas oryzae* [17] or *Campylobacter* [18]. In these pathogens, LPS lacks the O-antigen. Depending on their structure, bacteria can exhibit two morphologies: a smooth form (S-form LPS), in which LPS consists of three components, and a rough form (R-form LPS or lipooligosaccharides, LOS), in which LPS is deprived of the O-antigen [8]. Studies have shown that even slight modifications in LPS structure may alter its overall conformation and biological activity.

## 3. Structural Modifications of Endotoxin Act as a Defence Mechanism for Bacteria, Shielding Them from the Immune System’s Attacks

To survive in the host, bacteria must deceive the immune system’s arsenal. Bacteria have developed many strategies, one of which is making changes in the structure of LPS. This strategy allows pathogens to stay under the immune system’s radar, dampening its ability to respond effectively. Among various structural alterations, modifications of lipid A are the most extensively studied, as they have the most significant impact on immune recognition. In contrast, changes in the core oligosaccharide or O-antigen regions occur less frequently and are typically subtler in their effects. It has been found that bacteria deceive the immune system by changing the molecule’s overall charge by removing phosphates from the core and disaccharide backbone of lipid A [8], and removing the phosphate group from lipid A results in the formation of non-toxic and non-pyrogenic structures of LPS. The pathogens can also mask the negative charge of lipid A by adding chemical groups (such as Arap4N, GlcpN, 2-amino-ethyl groups). As a consequence, the resistance to the effectors of innate immunity increases [8].

Concealing the charge of lipid A helps protect bacteria from cationic anti-microbial peptides (CAMPs), which are part of the host’s innate immunity [19]. Bacteria capable of masking their charge include, for example, *Pseudomonas aeruginosa*, *Burkholderia species* [20], *Proteus mirabilis* [21], *Klebsiella pneumoniae* [22], *Yersinia pseudotuberculosis* [23], and *Salmonella typhimurium* [24]. The *S. typhimurium* modifies LPS not only by adding the Arap4N group but also the 2-aminoethylphosphate (PEtN). Another pathogen that can mask its negative charge through dephosphorylation and addition of a chemical group is *H. pylori* [25].

Another bacterial strategy involves altering the degree and pattern of acylation of the lipid A moiety. This may contribute to the LPS being undetected by host immune cells or a change in the induced cytokine response [26]. An excellent example of a bacterium that exhibits this defence mechanism is *Shigella flexneri*. The lipid A isolated directly from infected epithelial cells was three- or four-acylated, unlike the typical hexa-acylated form isolated from the bacteria cultured in broth. This modification has also affected the immune response induced through Toll-like receptor 4 (TLR4). Hexa-acylated forms strongly activate human TLR4, whereas tetra-acylated forms do not exhibit noticeable activation. LPS modified in this manner has an antagonistic effect on TLR4. Moreover, the LPS characterised by a lower degree of acylation can silence the inflammasome activation, as evidenced by low levels of secreted interleukin (IL) 1β [27,28,29]. Another interesting pathogen that modulates the degree of LPS acylation is *Yersinia pestis*. When this bacterium is grown at a temperature close to that of the host (37 °C), it expresses mainly tetra-acylated lipid A, while at temperatures 21–28 °C it expresses mainly hexa-acylated structures [29]. The following examples are *F. tularensis* and *H. pylori*, pathogens also characterised by tetra-acylated lipid A of LPS [30]. Interestingly, *H. pylori* synthesises the haxa-acylated but presents a tetra-acylated form [25,29].

The next mechanism enabling pathogens to evade immune detection involves the differentiation of LPS carbohydrate domains (antigenic variability). *Neisseria* spp. and nontypeable *H. influenzae* can express core oligosaccharide structures characterised by inter- and intrastrain heterogeneity. This is due to the existence of highly variable glycoforms formed by switching terminal sugar units (*Neisseria* spp.) [31,32] or the ability to express a variety of oligosaccharide extensions (*H. influenzae*) [31,33], within a common internal core region among strains of each species. Such modifications make these structures similar to or even the same as those in the host organism. An excellent example may be the changes introduced by nontypeable *H. influenzae*, which makes oligosaccharides identical to the carbohydrate part of glycosphingolipids in erythrocyte membranes [34]. Thus, pathogens can mimic structures found in host cells, making them invisible to the immune system. This critical property is attributed to the sialic acid in the LPS O-specific region, which occurs on the surface of mammalian cells [10,35]. Furthermore, the supramolecular structure, particularly its disruption, also influences biological activity. There are indications that the disaggregation of LPS into monomers reduces the toxin’s activity [36,37].

## 4. How Do Organisms Sense LPS?

**Figure 2:**
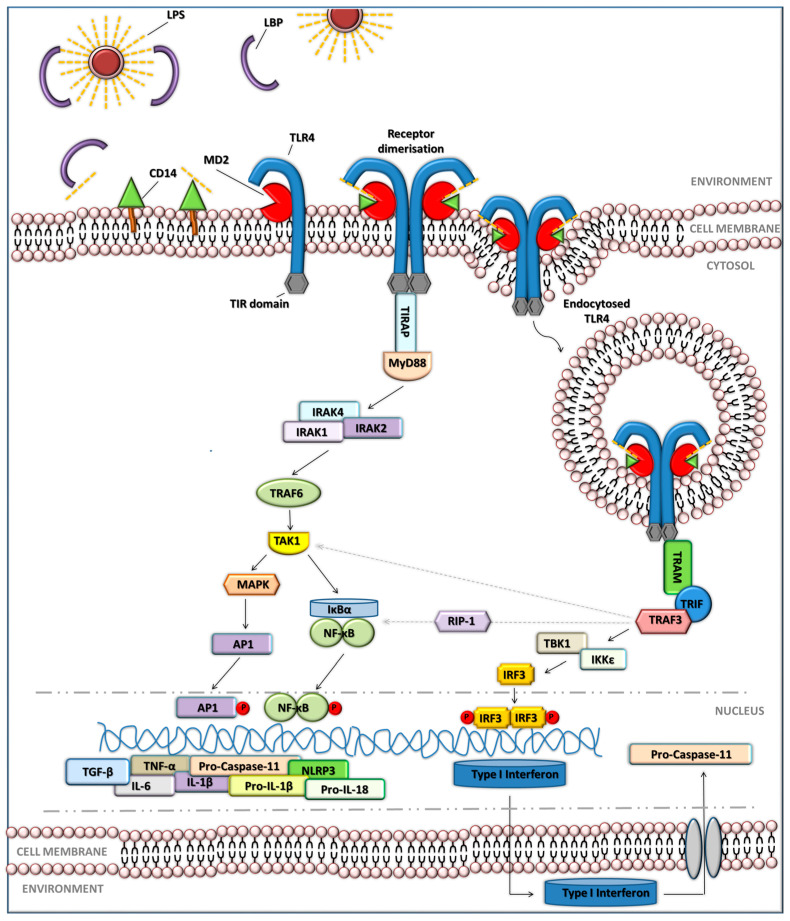
Toll-like receptor 4 signalling pathways. **On the cell surface (LPS recognition): LPS**—Lipopolysaccharide; **LBP**—LPS Binding Protein; **CD14**—Cluster of Differentiation 14; **MD2**—Myeloid Differentiation factor 2; **TLR4**—Toll-Like Receptor 4; **TIR domain**—Toll/Interleukin-1 Receptor domain; **TIRAP**—TIR domain-containing adaptor protein; **MyD88**—Myeloid Differentiation primary response 88. **MyD88-dependent signalling pathway: IRAK (1,2,4)**—Interleukin-1 Receptor-Associated Kinase (1,2,4); **TRAF6**—TNF Receptor-Associated Factor 6; **TAK1**—Transforming Growth Factor β-Activated Kinase 1; **MAPK**—Mitogen-Activated Protein Kinase; **AP1**—Activator Protein 1 transcription factor; **IκBα**—Inhibitor of κB α; **NF-κB**—Nuclear Factor κB; **RIP-1**—Receptor Interacting Protein kinase 1. **TRIF-dependent signalling pathway (endocytosed TLR4): TRAM**—TRIF-related adaptor molecule; **TRIF**—TIR-domain-containing adaptor inducing interferon-β; **TRAF3**—TNF Receptor-Associated Factor 3; **TBK1**—TANK-Binding Kinase 1; **IKKε**—IκB kinase ε; **IRF3**—Interferon Regulatory Factor 3. **Effector molecules and cytokines: TGF-β**—Transforming Growth Factor β; **TNF-α**—Tumour Necrosis Factor α; **IL-6**—Interleukin-6; **IL-1β**—Interleukin-1 β; **Pro-IL-1β**—precursor of interleukin-1 β; **Pro-IL-18**—precursor of interleukin-18; **NLRP3**—NOD-Like Receptor family Pyrin domain-containing protein 3; **Pro-Caspase-11**—precursor of caspase-11; **Type I Interferon**—Interferon type I (e.g., IFN-α, IFN-β). Dash line—late inflammation response.

Endotoxin, when introduced into the body, can lead to the activation of the innate immune response, primarily by activating the monocytes/macrophages, followed by the release of pro-inflammatory factors such as interferon (IFN) γ, tumour necrosis factor α (TNF-α), IL-6, IL-17, IL-1β, etc. [38]. The triggering of the inflammatory response due to the elevated level of LPS occurs due to the activation of the TLR4 signalling on the surface of phagocytic cells (Figure 2). In the signalling cascade, LPS, primarily occurring in micellar forms, binds to the LPS binding protein (LBP), which then facilitates the association between the monomer of LPS and cluster of differentiation (CD) 14. Followed by the transfer of LPS to the TLR4-myeloid differentiation protein 2 (MD2) complex on the cell surface. Once LPS is bound to TLR4-MD2, it induces a conformational change in TLR4, leading to the recruitment of downstream signalling molecules and activation of two pathways, i.e., the myeloid differentiation primary response gene 88 (MyD88)-dependent pathway and the MyD88-independent pathway [39,40].

In the MyD88-dependent pathway, the TIR domain of TLR4 first interacts with the Toll-interleukin-1 Receptor (TIR) domain-containing adaptor protein (TIRAP), which then facilitates the recruitment of the MyD88 adaptor protein. MyD88 subsequently binds to the TLR4-TIRAP complex, leading to the recruitment of interleukin-1 receptor-associated kinases (IRAKs) and tumour necrosis factor receptor-associated factor 6 (TRAF6), which in turn activate transforming growth factor-β-activated kinase 1 (TAK1) (Figure 2). Subsequently, this complex activates multiple signalling pathways, including the nuclear factor-κB (NF-κB) and the mitogen-activated protein kinase (MAPK) pathways. As a result of signalling events, the transcription and synthesis of pro-inflammatory cytokines, such as TNF-α, IL-1β, and IL-6, drive inflammation and recruit immune cells to the site of infection [41,42].

TLR4 signalling can also occur independently of the MyD88 adaptor molecule through an alternative pathway known as the TLR4-MyD88-independent pathway [43] (Figure 2). This pathway occurs after internalisation of the TLR4-MD2 complex and primarily relies on the recruitment of two additional adaptor molecules: TRIF-related adaptor molecule (TRAM) and Toll/IL-1 receptor domain-containing adaptor-inducing interferon-β (TRIF). TRIF then engages several downstream signalling molecules such as TNF receptor-associated factor 3 (TRAF3), TANK binding kinase 1 (TBK1), and IκB kinases (IKK), leading to the activation of distinct signalling pathways and the production of specific pro-inflammatory factors. One of the primary pathways activated by TRIF is the interferon regulatory factor 3 (IRF3) pathway. Activation of this pathway results in the phosphorylation and nuclear translocation of IRF3, which induces the transcription of genes encoding type I interferons (IFNs), including IFN-β. Type I IFNs play a crucial role in anti-viral defence and can also modulate the immune response by regulating the expression of other inflammatory genes [44,45,46].

In addition, TRIF can also activate the NF-κB pathway via receptor-interacting protein 1 (RIP1) and TRAF3, leading to the expression of pro-inflammatory cytokines such as TNF-α and IL-6 [47,48], as well as the MAPK pathway through TAK1, which phosphorylates downstream kinases like p38 MAPK, Jun N-terminal kinases (JNKs), and extracellular signal-regulated kinases 1/2 (ERK 1/2), further enhancing the inflammatory response [49,50,51].

## 5. Inflammasome Signalling

**Figure 3:**
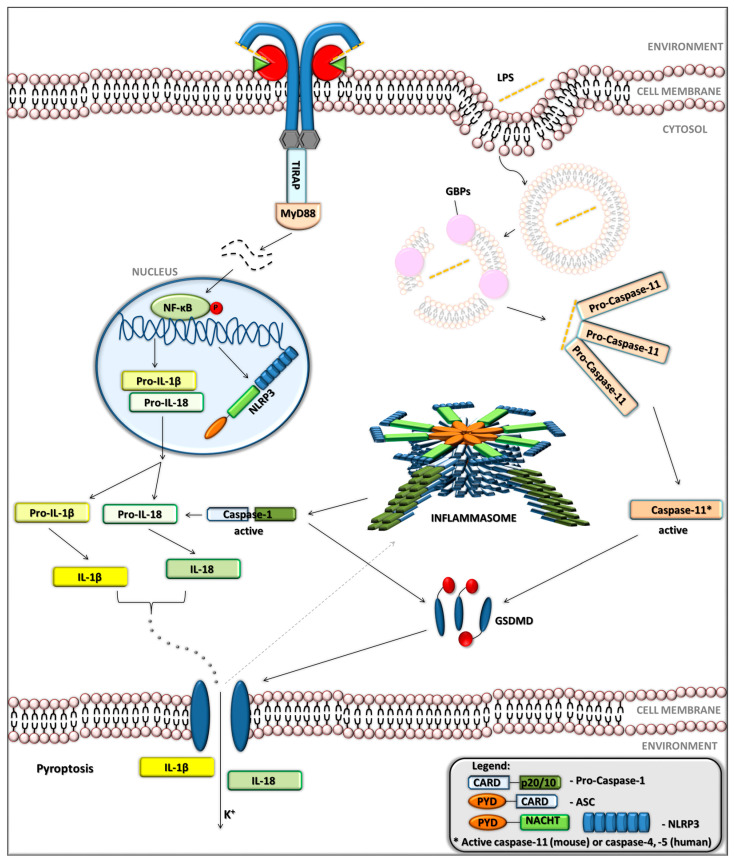
Schematic representation of the inflammasome signalling pathway, from priming and activation to cytokine maturation and pyroptotic cell death. **On the cell surface (LPS recognition): LPS**—Lipopolysaccharide; **TLR4**—Toll-Like Receptor 4; **TIRAP**—TIR domain-containing adaptor protein; **MyD88**—Myeloid Differentiation primary response 88. **Cytoplasmic sensors and regulators: NF-κB**—Nuclear Factor κB; **NLRP3**—NOD-Like Receptor family Pyrin domain-containing protein 3; **GBPs**—Guanylate-Binding Proteins. **Caspases and inflammasome: Pro-Caspase-11**—precursor of Caspase-11 (murine protease); Caspase-11*—active Caspase-11; **ASC**—Apoptosis-associated Speck-like protein containing a CARD; **GSDMD**—Gasdermin D. **Cytokines and precursors: Pro-IL-1β**—precursor of Interleukin-1 β; **Pro-IL-18**—precursor of Interleukin-18; **IL-1β**—Interleukin-1 β; **IL-18**—Interleukin-18. **Protein domains: CARD**—Caspase Activation and Recruitment Domain; **PYD**—Pyrin Domain; **NACHT**—Nucleotide-binding and oligomerization domain; **p20/p10**—Catalytic subunits of Caspase-1. Dashed line—feedback activation of the inflammasome.

Following the recognition of bacterial LPS by TLR4, a cascade of intracellular signalling is initiated, culminating in the activation of transcription factors such as NF-κB. This transcriptional response leads to the upregulation of numerous inflammatory mediators, including cytokines, chemokines, and components of the inflammasome machinery such as nucleotide-binding oligomerization domain (NOD)-like pyrin domain-containing protein 3 (NLRP3) [52,53]. IL-1β and IL-18 are particularly significant among the induced cytokines due to their potent pro-inflammatory activity. However, both are initially produced as inactive precursors (pro-IL-1β and pro-IL-18) and require subsequent proteolytic processing to become biologically active [52]. This processing is mediated by caspase-1, a protease activated within a multiprotein complex known as the NLRP3 inflammasome (Figure 3). The activation of this complex occurs via two pathways: the canonical and the non-canonical routes [53].

In the canonical pathway, TLR4-driven priming induces the transcription of NLRP3, pro-IL-1β, and pro-IL-18. Full activation of the canonical pathway requires the second signal, which is provided by stimuli such as extracellular ATP, pore-forming toxins, or crystalline particles. This leads to NLRP3 oligomerisation and assembly of a cytoplasmic complex composed of NLRP3, the adaptor protein ASC (apoptosis-associated speck-like protein containing a CARD), and pro-caspase-1. ASC and NLRP3 interact with pro-caspase-1 upon activation, leading to caspase-1 autoproteolytic activation [53]. Once active, caspase-1 cleaves pro-IL-1β and pro-IL-18 into their mature forms. Caspase-1 also cleaves gasdermin D (GSDMD), a pore-forming protein that facilitates cytokine release and induces pyroptosis, a lytic and inflammatory form of programmed cell death [54,55].

In parallel, the non-canonical pathway provides an alternative route of inflammasome activation that serves as a defence mechanism against pathogens that have developed strategies to evade TLR4 (Figure 3) [56,57]. In this case, cytosolic LPS is directly sensed by murine caspase-11 or its human homologs, caspase-4 and caspase-5 [58]. Guanylate-binding proteins (GBPs) facilitate LPS exposure and recognition in the cytoplasm. Once activated, caspase-11 cleaves GSDMD, initiating pore formation, potassium efflux, and ATP release. These ionic disturbances serve as secondary triggers for NLRP3 and caspase-1 activation. While caspase-11 does not directly process pro-inflammatory cytokines, it indirectly drives IL-1β and IL-18 maturation by enabling canonical inflammasome formation [59,60]. Thus, both canonical and non-canonical inflammasome pathways ultimately converge on caspase-1 activation, ensuring a robust and multi-layered inflammatory response. This integration of TLR4 signalling with inflammasome-mediated effector functions underscores the complexity and redundancy of the innate immune system in defending against microbial threats, particularly those that attempt to evade surface receptor recognition [61].

## 6. Acute Host Response to Endotoxin: Fever as a Hallmark of Endotoxin-Induced Inflammation

LPS triggers a rapid and robust acute inflammatory response driven by the activation of innate immune pathways. As mentioned above, engagement of receptors such as TLR4 leads to the release of pro-inflammatory cytokines like TNF-α, IL-1β, and IL-6. This cascade results in the classical hallmarks of acute inflammation, including leukocyte recruitment, increased vascular permeability, and fever. Among these, fever is one of the most recognisable and evolutionarily conserved responses to infection. Historical references to endotoxin date back to the 18th century, when it was observed that a substance derived from decaying organic matter could induce fever [8]. Today, fever is understood as a key physiological defence mechanism, driven by pro-inflammatory cytokines such as IL-1β, IL-6, and TNF-α, known as endogenous pyrogens. It reflects the host’s attempt to inhibit pathogen replication, enhance immune cell function, and restore homeostasis during the early stages of infection [62,63]. In endotoxemia, the febrile response plays a dual role: it contributes to pathogen clearance while simultaneously amplifying immune activation [62,64]. Elevated body temperature supports the efficiency of immune cells, including neutrophils and macrophages, and promotes the production of acute-phase proteins [64,65]. This complex interplay between endotoxins, cytokine release, and thermoregulation has been thoroughly explored in several review articles, to which readers are encouraged to refer for deeper insight into molecular mechanisms and clinical implications [62,64,65,66].

## 7. Beyond the Acute Response: Chronic Inflammation and Endotoxin Tolerance as Distinct Immune Outcomes of Endotoxin Exposure

### 7.1. Chronic Inflammation

While acute inflammation is a protective and self-limiting response to infection or injury, failure to resolve it properly can lead to a chronic inflammatory state. This transition may occur when the initial stimulus persists or regulatory mechanisms break down. Unlike in acute inflammation, where cytokine release is rapidly downregulated after pathogen clearance, LPS-driven chronic inflammation maintains a low-grade but persistent immune activation that disrupts tissue homeostasis. Over time, this state contributes to tissue damage, remodelling, and, in some cases, fibrosis [2,67].

Clinically, chronic inflammation induced by LPS is implicated in the development of several non-infectious diseases, including atherosclerosis, insulin resistance, and neurodegeneration [2,67,68]. In particular, metabolic endotoxemia, characterised by elevated circulating LPS due to increased gut permeability, has been linked to obesity-related chronic inflammatory disorders [2,68,69].

Specific Gram-negative pathogens have been directly linked to this process. *Helicobacter pylori* produces LPS variants that sustain gastric inflammation and are associated with peptic ulcer disease and gastric cancer [70], while *Porphyromonas gingivalis*, a keystone pathogen in periodontitis, generates structurally unique LPS that not only drives local oral inflammation but also contributes to systemic conditions such as atherosclerosis [71]. These examples underscore that both pathogen-derived and commensal-derived LPS can serve as potent triggers of chronic inflammation.

A major source of persistent LPS exposure is the gut microbiota. Several Gram-negative species, including *Escherichia coli*, *Enterobacter cloacae*, and *Bacteroides fragilis*, produce highly immunostimulatory LPS [72,73,74]. Circulating LPS primarily activates TLR4 on immune cells, leading to NF-κB-dependent cytokine release and establishing a state of low-grade systemic inflammation that promotes metabolic and cardiovascular disorders [75]. In particular, metabolic endotoxemia, defined by elevated LPS levels due to impaired intestinal barrier integrity, has been closely associated with obesity-related inflammatory conditions [76].

The composition of the gut microbiota strongly influences the magnitude of LPS-driven inflammation. Under homeostatic conditions, commensals such as *Bacteroides thetaiotaomicron* and *Faecalibacterium prausnitzii* maintain epithelial integrity, produce short-chain fatty acids, and thereby limit systemic dissemination of LPS [77]. In contrast, dysbiosis, often triggered by a high-fat diet, antibiotics, or aging, promotes the expansion of Gram-negative taxa and reduces barrier-protective species, leading to enhanced intestinal permeability and LPS translocation into the circulation [78,79]. Notably, not all LPS molecules exert the same pro-inflammatory effects: structural variations in lipid A among bacterial species result in differential TLR4 activation, which may explain why microbiota composition has such a profound impact on systemic immune responses [80]. Moreover, additional signalling pathways, including CD14 co-receptor engagement and NLRP3 inflammasome activation, further amplify cytokine cascades and sustain chronic inflammation [81].

Taken together, these findings suggest that both the presence of specific Gram-negative bacteria and the overall balance of the gut microbial ecosystem are critical determinants of chronic LPS-induced inflammation. Accordingly, therapeutic strategies that aim to modulate microbiota composition, restore barrier integrity, or inhibit LPS-TLR4 signaling are being actively investigated as promising approaches to reduce LPS-driven systemic inflammation [72,82].

### 7.2. Endotoxin Tolerance (ET)

If the inflammatory response to LPS remains uncontrolled, this can lead to significant tissue damage and the emergence of pathological conditions such as sepsis. To overcome this type of exaggerated response, a protective mechanism known as ET has been observed in the host body [83]. There is a great deal of confusion surrounding chronic inflammation and ET. It is important to emphasise that the host may exist in these two distinct physiological states. To facilitate the understanding of these differences, we present a comparison (Table 1).

The first reports regarding ET appeared in the 20th century when Paul Beeson (1946) documented this phenomenon through the febrile response in a rabbit with several injections of typhoid vaccine [84]. Greisman and Hornick (1975) also reported that the continuous administration of endotoxin in healthy individuals led to a decline in febrile and subjective toxic responses [85]. Over time, subsequent groups and studies have confirmed the existence of ET, demonstrated among others with a notable decrease in mortality rates among hosts administered a lethal dose of endotoxin following the activation of ET. Similarly, both in vivo and ex vivo models in a clinical experimental study involving 16 healthy male volunteers at an intensive care research unit demonstrated that plasma peaks of TNF-α, IL-6, IL-10, interleukin-1 receptor antagonist (IL-1ra), and transforming growth factor-β (TGF-β) were attenuated by 46%, 36%, 45%, 10%, and 14%, respectively, during the secondary dose of LPS [86].

Further studies on this intricate, tolerant immune response revealed TLR4 as a crucial factor in its regulation [87]. A downregulation of the pro-inflammatory cytokines such as TNF-α, IL-6, and IL-1β, which are produced through TLR4/MyD88 signalling, has been observed along with MAPK3 [88]. Furthermore, the study of ET and its influence on the MAPK family, which is known to be activated by TAK-1, has shown that phosphorylation of ERK 1/2, JNK, and P38 MAPK is downregulated in cells exhibiting ET [89]. On the other hand, the expression of the anti-inflammatory cytokines such as IL-10 and TGF-β appeared to be upregulated with low doses of LPS [90]. These tolerant macrophages are also characterised by increased phagocytosis (bacterial clearance) and do not mount a fever response [83].

The results indicate that TNF-α emerges as a pivotal indicator of ET due to its dual role in triggering inflammation and inducing tolerance upon repeated exposure [91]. Concurrently, cytokines such as IL-10 and TGF-β exert an opposing influence by regulating activated macrophages via IRAK3 and suppressor of cytokine signalling 1 (SOCS1) signalling pathways. SOCS1, in particular, dampens the NF-κB pathway post-LPS treatment, contributing to the establishment of tolerance [92].

Studies, like the one conducted by Lee et al., have elucidated the temporal dynamics of ET in vivo, particularly during severe sepsis. IRAK-M, a negative regulator of TLR signalling, peaks around 12 to 24 h post-sepsis, indicative of heightened tolerance which subsequently wanes [93].

Another factor that may mediate ET is the miRNA machinery. miR-181b has been reported to potentially participate in the mechanism of endotoxin-triggered inflammation by stimulating the NF-κB signalling pathway in vitro [39]. Additionally, miR-155 was observed to be upregulated by endotoxin stimulation in macrophages of mice in vitro and in vivo via directly controlling NF-κB transcriptional activity [94]. It binds to SOCS1 to promote inflammation [95]. Thus, the downregulation of these miRNAs can be related to the enhancement of ET.

Circular RNAs (circRNAs) represent another crucial element of the miRNA regulatory machinery. These circRNAs are formed through back-splicing, in which the 3′ end of an exon is covalently linked to the 5′ end, resulting in a closed circular structure. Accumulating evidence indicates that circRNAs contribute to the pathogenesis of a wide spectrum of human diseases, including aging-associated disorders, cancer, cardiovascular and metabolic diseases, osteoarthritis, stress responses, and viral infections [96]. Beyond their roles in disease, circRNAs have also been shown to regulate macrophage biology. For example, mcircRasGEF1B was reported to be essential for NF-κB activation and macrophage responses [97]. Similarly, circRNA microarray analyses of bone marrow-derived macrophages differentiated into pro- and anti-inflammatory phenotypes revealed that circ-Cdr1as may exert an anti-inflammatory effect, being significantly downregulated in pro-inflammatory macrophages but strongly upregulated in anti-inflammatory macrophages, thereby promoting M2 polarization. CircRNAs can also function as miRNA sponges, influencing the expression of downstream proteins. For instance, overexpression of circ17725 in RAW264.7 macrophages led to the downregulation of TNF-α, IL-1β, and matrix metalloproteinase-9 (MMP-9) through its interaction with the miR-4668-5p/FAM46C [98]. In addition, circRNA profiles within extracellular vesicles obtained from bronchoalveolar lavage fluid were found to be significantly altered following LPS or acid instillation in mice. Notably, LPS-induced circ25900 expression in alveolar macrophages was attenuated after prolonged endotoxin treatment, suggesting a role for circRNAs in the dynamics of endotoxin responses [99].

While circRNAs illustrate how post-transcriptional mechanisms shape macrophage and endotoxin responses, genetic variation in upstream pattern-recognition receptors such as TLR4 may determine the extent of LPS-induced inflammatory signalling. Polymorphisms in the TLR4 gene significantly influence host responses to endotoxin and may modulate the development of ET. Variants at +896 bp (TLR4/+896) and +1196 bp (TLR4/+1196) have been associated with systemic hyporesponsiveness to LPS, as individuals heterozygous for either variant exhibited markedly reduced inflammatory responses following inhaled LPS challenge [100]. Two common co-segregating missense mutations, Asp299Gly and Thr399Ile, are thought to underlie this phenotype. Functional studies demonstrated that the Asp299Gly mutation, but not Thr399Ile, disrupts TLR4-mediated signalling in THP-1 cells, while transfection with the wild-type allele restores responsiveness [101,102]. Interestingly, this blunted response appears to be selective, predominantly affecting the induction of IL-6 and cyclooxygenase (COX) 2 [103]. In vivo, mice carrying the Asp299Gly and Thr399Ile polymorphisms showed improved survival after lethal LPS challenge, and macrophages derived from these mice displayed impaired immunometabolic reprogramming, with reductions in both glycolysis and mitochondrial respiration [104]. Furthermore, the effect of TLR4 polymorphisms appears to depend on the origin of LPS: the Asp299Gly variant reduced pro-inflammatory cytokine production in response to LPS derived from *Salmonella*, but not *to Escherichia coli* LPS, highlighting that the disease risk associated with endotoxin exposure is shaped by both host genetics and microbial variation [105].

To explore the immunomodulatory consequences of ET, we evaluated macrophage phenotype and function following LPS stimulation [106]. Notably, our analysis revealed that monocyte-derived endotoxin-tolerant macrophages (Mo_ETs_) predominantly expressed CD80, rather than CD163, indicating polarisation toward an M1-like phenotype. These findings are consistent with prior observations by Peña et al. (2011) [107], who also reported a dissociation between pro-inflammatory cytokine expression and M2 surface markers such as CD206.

## 8. Two-Faced Nature of ET

In nature, phenomena often reveal themselves as multifaceted, exhibiting both advantageous and detrimental aspects depending on the context. This duality is echoed in numerous studies across PubMed, exemplified by entities like Tripartite Motif Containing 8 (TRIM8), characterised as a “molecule of duality” due to its roles as both an oncogene and a tumour suppressor gene [108]. Autophagy, another compelling example, showcases a dichotomy in its function, with the potential to either benefit or harm tumours [109,110]. These instances underscore the intricate balance inherent in biological systems, where understanding the dual nature of such factors is pivotal for unravelling their full implications. ET exemplifies a paradoxical phenomenon, acting as a double-edged sword in its impact.

### 8.1. “The Bright Face” of ET

ET can be beneficial by preventing excessive inflammation and tissue damage from various causes. Its positive effect has been demonstrated on liver ischemia–reperfusion (IR) damage, a condition accompanied by oxidative stress leading to cell damage and the development of inflammation. This local reaction is driven by cells of the innate immune system. Studies conducted in a rat model have shown that pre-treatment with endotoxin reduces liver IR and renal IR [111,112]. This is probably related to IRAK4-dependent downregulation of interorgan TNF-α expression. Moreover, ET also affects the development of neurodegenerative diseases. It has been shown that the development of ET in microglial cells may contribute to a positive factor for neuroprotective effects in the brain [113,114].

ET plays a complex role in immune modulation. However, the most dramatic manifestations are observed in sepsis. It is known that under normal conditions, the immune response is controlled and resolves once the threat is neutralised. However, in some cases, immune regulation fails, and a life-threatening condition known as sepsis may develop. This is characterized by a systemic inflammatory reaction, tissue damage, and potential multi-organ failure. A hallmark of sepsis is the excessive and uncontrolled release of pro-inflammatory cytokines, commonly called a cytokine storm, which can cause widespread harm to host tissues and organs [115]. Organisms may activate ET to mitigate this inflammatory response. If these regulatory capacities are exhausted or fail, we believe a chronic inflammatory state becomes established, which is pathological and should not be considered an adaptive response. Thus, by dampening inflammation, ET may protect against organ dysfunction, such as acute lung injury, acute kidney injury, and cardiovascular collapse, which are common complications of severe sepsis. Indeed, research findings suggest that ET plays a crucial role in reducing the inflammatory response in the host, ultimately increasing survival rates in murine models of polymicrobial sepsis [116]. Similarly, Melo et al., also reported that the combination of decreased lymphocyte cell death, increased cell growth, and the concurrent strengthening of T cell TH1 and TH2 responses (evidenced by TNF-α, IL-4, and IL-10) during LPS tolerance seems to represent a robust host reaction. This demonstrates a successful interaction between the innate and adaptive immune systems, resulting in improved cellular functionality and increased chances of survival amidst a severe polymicrobial septic threat [117].

In line with the above information, ET has been reported to be associated with the increase in Treg and Th17 lymphocytes in blood and spleen in mice subjected to caecal ligation and puncture (CLP) and thereby protecting the animal from the hyperimmune response [118]. Also, an increase in anti-inflammatory factors such as IL-10 and IL-4 has been associated with increased survival rate in the case of sepsis [119]. ET’s adaptive immune modulatory response was also demonstrated in vivo, as the tolerant mice survived a lethal dose of intestinal bacteria [120]. In a similar scenario, ET has also been reported to increase bacterial clearance through promoting the arrival of polymorphonuclear neutrophils (PMNs) at the site of the LPS challenge, and these PMNs also demonstrate enhanced neutrophil extracellular trap (NET)-forming ability [121]. Together, these factors are beneficial in preventing the secondary infections that are observed during sepsis.

### 8.2. “The Dark Face” of Endotoxin Tolerance—Increased Disease Susceptibility with a Focus on Cancer

While ET may offer protection against the severe effects of sepsis, in certain scenarios, it can also prove to be a harmful mechanism. Since ET may weaken the immune response, it may render the host more susceptible to recurrent infections [122]. One such clinical condition is severe acute pancreatitis (SAP), during which the levels of endotoxin and the ability of whole blood cells to release TNF-α upon stimulation by LPS fall somewhere between those observed in systemic inflammatory response syndrome (SIRS) and severe sepsis. It is believed that ET might represent one aspect of immune dysfunction that complicates the clinical course of SAP patients [123]. According to findings from a 2014 cohort study, immune dysfunction mediated by ET has been noted to persist throughout the clinical progression of the disease and is linked to its severity [124].

ET, defined by a suppressed response to endotoxin, has paradoxically been associated with worse outcomes in sepsis in some instances. Unlike the protective “bright face” of ET, the blunted ex vivo cytokine response may sometimes reflect sustained and harmful inflammation in vivo [125]. Notably, the ET signature has been reported to be present in sepsis patients as early as day 1 following admission to the intensive care unit, and this association persisted through day 3. During the later phase of sepsis, an imbalance between inflammation and immunosuppression, primarily driven by ET, results in uncontrolled infection alongside excessive immune suppression [124]. This observation correlates with an RNA sequence analysis performed in patients with sepsis, which indicated the presence of ET in the very early stage of sepsis in most patients [126]. Considering these facts, ET may offer benefits only in the early stages of sepsis. During this period, it can mitigate tissue damage caused by cytokine storms. However, as this immune-paralysed state persists, the likelihood of secondary infections increases.

Another example of a clinical condition that involves ET is periodontal disease. In periodontitis, biofilm can disturb the gingival tissues, causing a compromised epithelial barrier and the formation of periodontal pockets, which are then colonised by Gram-negative bacteria. This colonisation can subsequently result in endothelial damage and the release of bacterial products into the bloodstream (endotoxemia), reinforcing the association between periodontitis and septic shock syndrome [127]. The stimulation of pro-inflammatory cytokines (such as IL-6, IL-8, IL-1β, and TNF-α) by LPS derived from *P. gingivalis* was notably reduced in fibroblasts isolated from inflamed gingiva affected by periodontitis compared to fibroblasts from healthy individuals, indicating heightened tolerance in the diseased state [128].

Reportedly, human periodontal ligament cells (hPDLCs) undergo a “negative regulation of immune response” as a result of repeated exposure to pathogens, therefore indicating the induction of endotoxin tolerance by LPS, resulting in the suppression of TLRs and the production of inflammatory cytokines [83].

**Figure 4:**
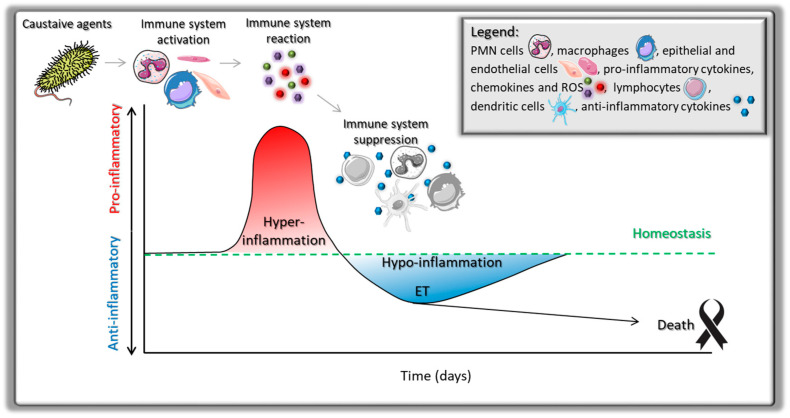
Schematic representation of types of immune response to Gram-negative bacteria regarding homeostasis.

Following exposure to bacterial endotoxins, the host immune response typically progresses through two distinct phases. The initial stage is characterized by a hyper-inflammatory reaction, during which activated neutrophils, macrophages, dendritic cells, and epithelial or endothelial cells release large amounts of pro-inflammatory cytokines, chemokines, and reactive oxygen species. While this robust response is critical for pathogen clearance, its excessive amplification may cause severe tissue damage [129,130]. To counteract such detrimental effects, the immune system subsequently enters a state of immune suppression, commonly referred to as ET. In this phase, immune cells exhibit reduced responsiveness to repeated LPS stimulation, producing lower levels of pro-inflammatory mediators while enhancing the release of anti-inflammatory cytokines [131,132,133]. Although ET serves as a protective mechanism against overwhelming inflammation, persistent hypo-responsiveness may impair microbial clearance, increase vulnerability to secondary infections, and ultimately compromise host survival [134,135]. Thus, the transition from hyper-inflammation to hypo-inflammation reflects a delicate balance between host protection and immune paralysis, with restoration of homeostasis being essential for recovery [133,136]. (Figure 4).

Adverse outcomes associated with ET extend beyond infections alone. Since a downregulation of pro-inflammatory responses characterises ET, a diminished febrile reaction is one of its hallmark manifestations. Interestingly, cancer patients have been observed to develop fewer and less intense fevers during infections compared to healthy individuals [137]. This clinical observation suggests a potential link between ET and impaired immune responses in cancer patients, possibly contributing to tumour progression by dampening protective inflammatory mechanisms such as fever. Fever has long been considered a natural anti-tumour response, with historical reports dating back to the early 19th century documenting spontaneous tumour remissions following febrile infections [138]. Notably, Dr. William Coley, the father of cancer immunotherapy, used bacterial extracts to induce fever in cancer patients, achieving remarkable outcomes. More recently, Wrotek et al. reported a case of stage IV melanoma in which the onset of fever significantly altered the course of the disease, resulting in remission or sustained stabilization [139].

In light of these observations, we investigated the direct impact of ET on cancer development using experimental models [106]. To better understand the role of ET in cancer biology, we employed an experimental model mimicking the tumour microenvironment (TME). This model assessed the functional impact of conditioned medium (CM) derived from endotoxin-tolerant macrophages (Mo_ETs_) on tumour cell behaviour. Specifically, we evaluated cancer cell survival, clonogenicity, and migratory capacity following CM stimulation. Additionally, we included a 3D spheroidal cancer model, which better reflects the physiological characteristics of solid tumours, including cell–cell and cell–matrix interactions, as well as structural resistance to therapies [140,141]. Our results demonstrated that CM derived from Mo_ETs_ significantly enhanced breast and colon cancer cells’ survival, colony formation, and migration. These findings suggest that ET may contribute to a tumour-supportive microenvironment. In line with previous studies [87,142], macrophages in an endotoxin-tolerant state displayed a pro-tumorigenic profile, marked by the suppression of pro-inflammatory cytokines and an enhanced expression of anti-inflammatory mediators. Interestingly, ET exhibited distinct effects depending on the tumour type. While Mo_ET_-conditioned medium enhanced growth and migration in breast cancer spheroids, no significant effect was observed in colon cancer spheroids. This discrepancy may be attributed to the chronic exposure of colon epithelial cells to bacterial LPS in the gut, which could lead to an adaptive desensitisation or altered responsiveness to further endotoxin-related signals [143,144,145].

Further investigation into the immunological crosstalk between Mo_ET_ and tumour cells revealed that co-culture systems of Mo_ETs_ with cancer cells resulted in a marked reduction in pro-inflammatory cytokine expression [106]. This observation suggests that ET contributes to establishing an immunosuppressive tumour microenvironment, which may facilitate tumour immune evasion and progression.

To better understand how Mo_ETs_ function within the tumour microenvironment, it is crucial to investigate how soluble factors released by cancer cells influence their phenotype and activity. Evaluating their interaction with tumour-derived signals could help determine whether ET contributes to tumour immune evasion and progression. Mo_ET_ behaviour was analysed concerning their production of pro-inflammatory mediators, metabolic profile, and phenotypic markers. Particular attention was given to the expression of key inflammation-related markers, including CD14 and COX-2. CD14, a co-receptor of TLR4, is central to LPS recognition [146,147], while COX-2 is an enzyme involved in the synthesis of pro-inflammatory prostaglandins [148,149]. Consistent with the observations of Martin et al. (2001) [150], CD14 levels in Mo_ETs_ remained unchanged. In contrast, COX-2 expression was significantly reduced. Furthermore, it was found that Mo_ETs_ produced significantly lower NO levels following exposure to cancer-derived factors when compared to control macrophages treated once with LPS (Mo_LPS_). This decrease was accompanied by reduced expression of inducible nitric oxide synthase (iNOS), the enzyme responsible for NO generation [151,152]. Given the observed suppression of NO, the production of reactive oxygen species (ROS), another key mediator of inflammation, was measured. The analysis revealed that Mo_ET_ exhibited elevated levels of ROS, a result consistent with previous studies linking increased ROS production to the development of a pro-tumourigenic environment [153,154]. These findings further support the notion that Mo_ET_ contributes to establishing a local immune milieu that favours tumour progression.

In addition to functional characteristics, the survival of Mo_ETs_ in the tumour microenvironment was evaluated. Mo_ETs_ demonstrated increased viability following stimulation with CM derived from breast cancer cells, compared to control macrophages treated with LPS only once. These results imply that ET may enhance macrophage persistence within the tumour niche, supporting an immunosuppressive landscape facilitating tumour progression [124].

Notably, ET macrophages exhibited unique metabolic characteristics within the tumour context. SCENITH analysis indicated that Mo_ETs_ possess considerable metabolic flexibility [155]. While these cells rely heavily on glycolysis, a pathway widely recognised for supporting tumour growth [156,157], they also appeared to engage compensatory metabolic programs in response to inhibition of oxidative phosphorylation (OXPHOS). Although OXPHOS is typically linked to M2 macrophage polarisation [158], our findings suggest that Mo_ETs_ may shift between pathways to maintain energy homeostasis and survival. This adaptability may underlie their functional resilience within the metabolically demanding tumour microenvironment.

## 9. Clinical and Therapeutic Applications Related to Endotoxin

Endotoxins play a pivotal role not only in bacterial pathogenesis but also in shaping therapeutic strategies against excessive inflammation. Given their ability to trigger potent immune responses, endotoxins are both a clinical threat, especially in conditions like sepsis, and a potential tool in immunotherapy.

### 9.1. Sepsis and Anti-Endotoxin Strategies

As outlined in previous sections, severe Gram-negative bacterial infections often lead to endotoxemia and septic shock, characterised by uncontrolled systemic inflammation. Therapeutic efforts have focused on neutralising LPS or blocking its interaction with host receptors. Strategies include the use of endotoxin-neutralising agents such as polymyxins, TLR4 antagonists, and extracorporeal endotoxin removal techniques like polymyxin B hemoperfusion. Among the polymyxins used in medical practice are polymyxin B and colistin (polymyxin E), which are cationic lipopeptide antibiotics, produced by Gram-positive bacteria of the genus *Penibacillus*. The mechanism of action of these molecules is based on the destabilisation of the bacterial outer membrane, leading to bacterial cell death [159,160]. Polymyxins interact with LPS through positively charged α,γ-diaminobutyric acid residues, which bind to the anionic phosphate groups of LPS, leading to perforation of the bacterial cell membrane [159,160]. For this reason, these substances constitute antibiotics targeted at killing Gram-negative bacteria with a limited effect on Gram-positive ones [160]. Due to their ability to bind LPS and exhibit anti-microbial activity, these substances have shown potential for treating bacterial infections and LPS detoxification. However, the use of polymyxin antibiotics has been identified to have strong neurotoxic and nephrotoxic adverse side effects, which limit the application of these drugs. According to a retrospective cohort study performed by Aysert-Yildiz et al., neurotoxicity and nephrotoxicity are common among patients treated with polymyxin B and colistin. Of the 147 patients enrolled in the neurotoxicity tests, 13 of 77 and 1 of 70 showed symptoms of neurotoxicity after polymyxin B and colistin administration, respectively. Additionally, among 290 patients, 44.7% and 40.0% developed acute kidney injury, leading in many cases to withdrawal of treatment using these drugs [161]. Due to such complications related to polymyxin administration, an alternative approach—polymyxin B hemoperfusion cartridge (PMX-HP)—has emerged to remove LPS from the bloodstream. This technique uses polymyxin B immobilised on polystyrene fibers [162,163]. The mechanism of action is based on blood filtration using cartridges filled with adsorptive material, which captures harmful LPS, allowing purified blood to pass through. The covalent bond between fiber and polymyxin B prevents possible detachment and entry of polymyxin B into the bloodstream, reducing the presence of adverse events during the procedure related to the neurotoxic and nephrotoxic nature of this substance [162,163]. According to a meta-analysis performed by Li et al. (2010), application of PMX-HP in sepsis treatment reduces overall mortality among less severe cases compared to conventional therapies, which may result from decreased endotoxin level in the blood [163]. Additionally, in their network meta-analysis, Chen et al. (2023) pointed out the possible beneficial effect of PMH-HP usage in treating severe infections or sepsis among adult patients [164]. Antagonists of TLR4 are molecules that function as inhibitors of TLR4 signalling pathways to treat endotoxemia, sepsis and septic shock. Interaction of such molecules with these receptors results in blockage of the extracellular domain binding site for LPS, leading to reduced cellular responsiveness to endotoxin. Blockage of the TLR4 signalling pathway inhibits the expression of pro-inflammatory factors that determine the severity of these diseases. Two TLR4 antagonists—eritoran and resatorvid (TAK-242)—were enrolled in clinical trials focused on sepsis and septic shock treatment. However, neither expresses significant results in sepsis treatment [165,166,167]. Although some approaches showed promise in preclinical models, clinical trials have yielded mixed results, underlining the complexity of translating LPS-targeted therapies into effective treatments.

### 9.2. LPS as a Vaccine Adjuvant

Paradoxically, the immunostimulatory properties of LPS have been harnessed in vaccine development. Intensive research has led to the development of LPS analogues that have found application as vaccine adjuvants, some of which have even been approved for use (Table 2). Monophosphoryl lipid A (MPL), a detoxified derivative of LPS from *Salmonella minnesota*, retains the ability to activate immune responses through TLR4 but with reduced toxicity [168,169,170,171]. MPL is approved as an adjuvant in several human vaccines, such as the human papillomavirus (HPV), hepatitis B virus (HBV), Herpes Zoster, malaria and respiratory syncytial virus (RSV) [169,170]. The toxicity of MPL is approximately 1000-fold lower than that of LPS of the same origin [169,171]. It is assumed that the reduced cytotoxicity of MPL is a result of a combination of two factors: activation of the TRIF-dependent pathway over the MyD88-dependent pathway, which occurs after LPS binding to TLR4, and the presence of a heterogeneous mixture of lipid A congeneric forms having different numbers of acyl chains. Those forms containing five and six acyl chains were found to be TLR4 agonists, whereas three and four antagonists lowered the toxicity of MP [170]. MPL is also a component of more complex vaccine adjuvants known as AS01, AS02 and AS04. The first two are combinations of MPL with QS21, a triterpene glycoside isolated from *Quillaja saponaria*, which are formulated in cholesterol-containing liposomes and squalene oil-in-water emulsion, respectively [168,169,170]. In the context of AS04 adjuvant, it contains MPL adsorbed onto aluminium, which prolongs cytokine production after injection [168]. At the moment, there are at least five licensed vaccines, including the mentioned adjuvants: Fendrix^®^, Cervarix^®^, Shingrix^®^, Arexvy^®^, and Mosquirix^®^ [169,170,172]. Fendrix^®^ was the first vaccine to include MPL as an adjuvant component, which was approved and officially licensed. In this case, MPL was composed within the AS04 adjuvant system [169]. This vaccine, produced in 2004, contains AS04 with the recombinant surface antigen sAg of the HBV, against which it was intended to confer immunity [172]. The same adjuvant was used in Cervarix^®^, the vaccine produced to protect against 16 and 18 HPV strains. For this vaccine, AS04 was added to virus-like particles of the HPV L1 epitope of this virus [172]. Arexvy^®^, Shingrix^®^ and Mosquirix^®^ contain AS01 adjuvant [169,171,172]. Shingrix^®^ is a vaccine against Herpes Zoster, based on recombinant glycoprotein E of *Varicella zoster*. Arexvy^®^ was created in order to treat RSV infections. This vaccine include trimeric pre-fusion form of the RSV F-glycoprotein [170,173]. Mosquirix^®^ is a vaccine developed to prevent malaria, with the circumsporozoite protein as its main component [169,172]. Not only is MPL used in vaccines, but also its modified forms, such as glucopyranosyl lipid A (GLA) and second-generation lipid adjuvant (SLA). GLA, also known as phosphorylated hexaacyl disaccharide (PHAD), is a synthetic analogue of lipid A characterised by a hexa-acyl structure, improving adjuvant properties [169,170,171]. This substance is currently being clinically tested as an adjuvant for vaccines against human immunodeficiency virus (HIV), influenza, tuberculosis, schistosomiasis, RSV and malaria, with trials currently in Phase I and II [169,170]. SLA is a modified form of GLA, with truncated side lipid chains. This modification increases affinity to the hydrophobic pocket in MD2, resulting in increased immunological response [170,171]. Currently, Phase I and Phase II clinical trials with this adjuvant are being conducted, testing vaccines against Leishmaniasis and Herpes Zoster [170]. These many modifications of LPS used as adjuvants support the “bright face” of endotoxin in immunomodulation, leaving the door open for further research in this direction.

### 9.3. Endotoxin as a Biomarker and Diagnostic Tool

Circulating levels of LPS can serve as a biomarker for diagnostic inflammation and infection-related diseases. There are many techniques of LPS determination, such as the rabbit pyrogen test, *Limulus* amebocyte lysate assay, endotoxin activity assay, recombinant factor C assay, bovine whole blood assay, monocyte activation test, ELISA-based assays, and mass spectrometry [174,175,176,177,178]. However, measurement of LPS concentration in the bloodstream is not commonly used in clinical practice, even for conditions such as sepsis or septic shock, which pathogenesis is usually directly associated with this toxin [179,180]. There are several drawbacks to using LPS as a biomarker, significantly limiting the applicability of this measurement in clinical practice. LPS is a molecule that readily undergoes modifications and varies between different bacterial strains of origin, substantially reducing its detectability [181]. Additionally, LPS can mask its presence in the bloodstream via interactions with many proteins, lipoproteins and salts, leading to inaccuracies in the quantification of free-circulating LPS [176,182]. Furthermore, techniques used to measure LPS have minuses, limiting their applicability in medical practice [174,175,176,177]. However, some studies indicated that LPS can function as a disease indicator. A meta-analysis performed by Soppert et al. shows that LPS concentration in peripheral blood is elevated during nonalcoholic fatty liver disease (NAFLD). According to 34 other studies, analysis shows that endotoxin levels rise with the severity of the liver steatosis and fibrosis. In their study, steatosis patients expressed an increased LPS level compared to healthy participants. The same correlation was observed between patients with nonalcoholic steatohepatitis and those with nonalcoholic fatty liver [183]. Teixeira et al. study indicates that an increased endotoxin level is also observed among COVID-19 patients. This study enrolled 66 COVID-19-positive and 6 control patients to assess whether microbial translocation markers are associated with systemic inflammation and may contribute to mortality in hospitalised COVID-19 patients. Based on their outcomes, infected patients expressed an increased level of LPS in blood samples, which significantly increased for the non-survivors group. In addition, markers of microbial translocation were upregulated compared to the control group [184]. Those and many other studies highlight LPS as a possible biomarker of intestinal permeability during conditions with ongoing inflammation. It may influence bacteria and endotoxin translocation to peripheral blood, increasing their level in the bloodstream [183,184,185,186,187,188,189]. The broad applicability of LPS determination as a biomarker in various conditions raises concerns regarding its specificity as a diagnostic indicator. Additionally, measurement techniques that lack sufficient efficiency and precision strongly reduce the possibility of using this substance as a biomarker. The development of new analytical techniques would be necessary to use this parameter. Nevertheless, monitoring LPS levels may help guide treatment decisions and predict outcomes in chronic inflammatory diseases associated with gut permeability and, consequently, progression of endotoxemia.

## 10. Conclusions

Endotoxin, such as LPS, is a central modulator of host immune responses to Gram-negative bacteria and remains a pivotal focus of research for elucidating the balance between protective immunity and immunopathology. Depending on the context, endotoxin can induce a wide range of immune outcomes: it may initiate an acute inflammatory response essential for effective pathogen clearance; it can drive chronic low-grade inflammation, contributing to tissue damage and disease progression; or it may lead to endotoxin tolerance, a hyporesponsive state that can be either protective or deleterious. Elucidating the molecular and cellular determinants that govern whether LPS exposure leads to immune activation, chronic persistence, or tolerance is essential for developing targeted therapies capable of precisely modulating immune responses. This also highlights that interpreting the clinical significance of elevated endotoxin levels requires a comprehensive understanding of the patient’s overall immunological and physiological context, since in some cases endotoxin presence may be directly harmful, while in others it may have triggered adaptive mechanisms that protect against severe outcomes or death.

## Figures and Tables

**Table 1:** Contrasting endotoxin tolerance and chronic inflammation mechanisms.

Stimulation	Chronic Inflammation	Endotoxin Tolerance
The alarm goes off (LPS)	The system keeps running and will not shut down	The system has “overheated” and stopped responding
Immunological outcome	Ongoing battle, tissue destruction	Immune silence due to hyporesponsivenessbut at the cost of vulnerability to other threats such as new infections or secondary pathogens
Adaptive function	Low—destructive and uncontrolled response	High—protective mechanism against immunopathology
Mechanism responsible for lack of fever	Low-level immune activation that does not reach the threshold needed to trigger a robust febrile response	Functional reprogramming of innate immune cells leading to sustained suppression of fever, even after repeated LPS exposure, unless tolerance is overcome

**Table 2:** Summary of various LPS analogues as components of adjuvants currently used in clinical studies [169,170,171].

Type of LPS Analogues	Advantage of LPS Modification	Adjuvant	Composition	Conditions	Phase of Development
MPL	Reduced toxicity in comparison to LPS	AS01	MPL with QS21 in liposome form	Malaria Herpes Zoster RSV	Approved Approved Approved
		AS02	MPL with QS21 in squalene oil-in-water emulsion	HIV Tuberculosis Hepatitis B	I II III
		AS04	MPL adsorbed onto aluminium	HBV HPV	Approved Approved
GLA	Increased immunological response compared to MPL. Synthetic substance.	GLA-AF	GLA in an aqueous form	HIV Influenza Schistosomiasis Hookworm	I I I I
		GLA-LSQ	GLA with QS21 in liposome form	Malaria Plasmodium Falciparum	I I
		GLA-SE	GLA in squalene oil-in-water emulsion	Schistosomiasis Leprosy Tuberculosis Influenza Malaria HIV Leishmaniasis RSV	II I II II I I I II
SLA	Increased immunological response compared to GLA. Synthetic substance.	SLA-SE	SLA in squalene oil-in-water emulsion	Leishmaniasis Herpes Zoster	I II

## Data Availability

All figures are original.

## Abbreviations

The following abbreviations are used in this manuscript:

ASC	Apoptosis-associated speck-like protein containing a CARD
CAMPs	Cationic anti-microbial peptides
CLP	Caecal ligation and puncture
CM	Conditioned medium
COX	Cyclooxygenase
ERK 1/2	Extracellular signal-regulated kinases 1/2
ET	Endotoxin tolerance
GBPs	Guanylate-binding proteins
GLA	Glucopyranosyl lipid A
GSDMD	Gasdermin D
HBV	Hepatitis B virus
HIV	Human immunodeficiency virus
hPDLCs	Human periodontal ligament cells
HPV	Human papillomavirus
IFN	Interferon
IL	Interleukin
IL-1ra	Interleukin-1 receptor antagonist
iNOS	Inducible nitric oxide synthase
IR	Ischemia–reperfusion
IRAK	Interleukin receptor-associated kinase
IRF3	Interferon regulatory factor 3
JNK	Jun N-terminal kinase
LBP	LPS binding protein
LOS	Lipooligosaccharide
LPS	Lipopolysaccharide
LRR	Leucine-rich repeat
MAPK	Mitogen-activated protein kinase
MD2	Myeloid differentiation protein 2
Mo_ETs_	Monocyte-derived endotoxin-tolerant macrophages
Mo_LPS_	Monocyte-derived endotoxin-treated macrophages
MPL	Monophosphoryl lipid A
MyD88	Myeloid differentiation primary response gene 88
NAFLD	Nonalcoholic fatty liver disease
NET	Neutrophil extracellular trap
NF-κB	Nuclear factor-κB
NLRP3	Receptors and pyrin domain-containing protein 3
NOD	Nucleotide-binding oligomerization domain
OXPHOS	Oxidative phosphorylation
PAMP	Pathogen-associated molecular pattern
PEtN	2-aminoethylphosphate
PHAD	Phosphorylated hexaacyl disaccharide
PMN	Polymorphonuclear neutrophil
PMX-HP	Polymyxin B hemoperfusion cartridge
RIP1	Receptor-interacting protein 1
ROS	Reactive oxygen species
RSV	Respiratory syncytial virus
SAP	Severe acute pancreatitis
SIRS	Systemic inflammatory response syndrome
SLA	Second-generation lipid adjuvant
SOCS1	Suppressor of cytokine signalling 1
TAK1	Transforming growth factor-β-activated kinase 1
TBK1	TANK binding kinase 1
TGF-β	Transforming growth factor-β
TIR	Toll-interleukin-1receptor
TIRAP	TIR domain-containing adaptor protein
TLR4	Toll-like receptor 4
TME	Tumour microenvironment
TNF-α	Tumor necrosis factor α
TRAF3	TNF receptor-associated factor 3
TRAF6	Tumour necrosis factor receptor-associated factor 6
TRAM	Toll/IL-1 receptor domain-containing adaptor-inducing IFN-β-related adaptor molecule
TRIF	Toll/IL-1 receptor domain-containing adaptor-inducing interferon-β
TRIM8	Tripartite motif containing 8
